# Equalization enhanced phase noise in Nyquist-spaced superchannel transmission systems using multi-channel digital back-propagation

**DOI:** 10.1038/srep13990

**Published:** 2015-09-14

**Authors:** Tianhua Xu, Gabriele Liga, Domaniç Lavery, Benn C. Thomsen, Seb J. Savory, Robert I. Killey, Polina Bayvel

**Affiliations:** 1 Optical Networks Group, Department of Electronic & Electrical Engineering, University College London, London, WC1E 7JE, United Kingdom

## Abstract

Superchannel transmission spaced at the symbol rate, known as Nyquist spacing, has been demonstrated for effectively maximizing the optical communication channel capacity and spectral efficiency. However, the achievable capacity and reach of transmission systems using advanced modulation formats are affected by fibre nonlinearities and equalization enhanced phase noise (EEPN). Fibre nonlinearities can be effectively compensated using digital back-propagation (DBP). However EEPN which arises from the interaction between laser phase noise and dispersion cannot be efficiently mitigated, and can significantly degrade the performance of transmission systems. Here we report the first investigation of the origin and the impact of EEPN in Nyquist-spaced superchannel system, employing electronic dispersion compensation (EDC) and multi-channel DBP (MC-DBP). Analysis was carried out in a Nyquist-spaced 9-channel 32-Gbaud DP-64QAM transmission system. Results confirm that EEPN significantly degrades the performance of all sub-channels of the superchannel system and that the distortions are more severe for the outer sub-channels, both using EDC and MC-DBP. It is also found that the origin of EEPN depends on the relative position between the carrier phase recovery module and the EDC (or MC-DBP) module. Considering EEPN, diverse coding techniques and modulation formats have to be applied for optimizing different sub-channels in superchannel systems.

The global demand for communication capacity is continuously growing, driven by the growth of mobile, video and cloud services and machine-to-machine communications^1^. Over 95% of this traffic is carried over optical fibres with a new challenge in developing optical fibre communication systems with increased capacity and spectral efficiency^2^,^3^. Traditionally viewed as having limitless capacity, the challenge of meeting the growing capacity demands have driven research towards techniques of accessing the fibre capacity in the most efficient way possible. One approach is to increase the use of the optical fibre spectrum (known as spectral efficiency) using a large number of closely-spaced wavelength channels. By using phase-locked optical sub-carriers, each sub-channel can be modulated at the highest possible level of modulation order, spaced at the symbol rate or near to the symbol rate, to generate a Nyquist or quasi-Nyquist spaced “superchannel” system^4^,^5^,^6^,^7^. This is combined with coherent optical detection, which gives access to both the received signal amplitude and phase, with many of the effects impairing quality of transmission, such as chromatic dispersion (CD), polarization mode dispersion (PMD), laser phase noise and fibre nonlinearities, which can be effectively fully or partially compensated through the use of digital signal processing (DSP)^8^,^9^,^10^,^11^,^12^,^13^,^14^. It has been demonstrated that carrier phase estimation (CPE) and digital back-propagation (DBP) DSP algorithms are effective for compensating both laser phase noise and nonlinear Kerr effects in optical fibre transmission systems^12^,^13^,^14^,^15^,^16^,^17^,^18^,^19^,^20^,^21^,^22^. However, in dispersion-unmanaged transmission system, laser phase noise can be converted into amplitude noise, due to the phase modulation to amplitude modulation (PM-AM) conversion induced by the group velocity dispersion in the fibre^23^,^24^. In DSP based coherent system, this phenomenon leads to an interaction between laser phase noise and electronic dispersion compensation (EDC), inducing an effect of equalization enhanced phase noise (EEPN)^25^,^26^. The EEPN has already been identified as a source of significant degradation in the performance of single-channel transmission systems, increasing with fibre dispersion, LO laser linewidth, modulation format and symbol rate^25^,^26^,^27^,^28^,^29^,^30^,^31^,^32^,^33^,^34^.

However, to date the impact of EEPN has not been investigated in multi-channel (including superchannel) systems. Yet, in multi-channel transmission, the effects of fibre nonlinearities are more significant as the channel spacing is decreased to Nyquist-spacing. Compared to conventional single-channel DBP for self-phase modulation (SPM) compensation, considerable benefits can be obtained by applying multi-channel DBP (MC-DBP) over the entire superchannel bandwidth^18^,^19^,^20^,^21^,^22^. This allows to compensate inter-channel nonlinear effects such as cross-phase modulation (XPM) and four-wave mixing (FWM) across the entire superchannel. MC-DBP can also be operated at different digital bandwidths, involving different numbers of sub-channels to achieve a compromise between the performance improvement and the computational complexity^21^,^22^. Several factors influencing superchannel transmission, MC-DBP, and nonlinear pre-compensation have been studied, such as the impact of nonlinear signal-noise interaction, accumulated PMD, comb carrier frequency uncertainty and LO phase imperfect synchronization^35^,^36^,^37^,^38^. However, to date, no investigation of the effects of EEPN on the performance of superchannel transmission and MC-DBP has been reported. In fact, EEPN may significantly distort the performance of such schemes, especially when the chromatic dispersion must be simultaneously compensated over the entire superchannel bandwidth, such as in the case of the use of full-bandwidth DBP. The impact of EEPN may differ for different sub-channels within the same superchannel, which must be taken into consideration for the optimization of optical fibre networks. Therefore, it is of both great practical importance and interest to study the impact of EEPN on the performance of superchannel transmission systems, for both EDC and MC-DBP.

In this paper, we investigate, for the first time to the best of our knowledge, the origin and influence of EEPN on the performance of the long-haul Nyquist-spaced superchannel transmission system, with and without the use of MC-DBP. Analysis has been carried in a Nyquist-spaced 9-channel 32-Gbaud dual-polarization 64-QAM (DP-64QAM) wavelength division multiplexing (WDM) superchannel system, with a total raw capacity of 3.456-Tbit/s. The achievable transmission distance of this superchannel system was evaluated both numerically, using the split-step Fourier algorithm, and analytically, using the perturbative Gaussian noise (GN) model. The performance of each sub-channel in the 9-channel DP-64QAM superchannel transmission system was investigated in detail to assess the origin and the influence of EEPN. Our results indicate that with both EDC and MC-DBP, EEPN causes a significant deterioration in the performance of all sub-channels, with penalties more severe for the outer sub-channels. Meanwhile, it is also shown that the source of EEPN, from the transmitter (Tx) laser or the local oscillator (LO) laser, depends on the relative position between the carrier phase estimation and the MC-DBP (or the EDC) modules.

Figure 1 schematically shows the origin of EEPN in long-haul coherent optical communication systems, in which electronic CD post-compensation and carrier phase estimation are employed. In Fig. 1, two scenarios are considered within the DSP modules: (a) CD compensation applied prior to CPE, (b) CD compensation applied after CPE. Generally, scenario (a) is more common for DSP operation in digital coherent receivers, since the required minimum oversampling rate (1 sample/symbol) in the CPE operation is less than that in the EDC and the MC-DBP operations and some adaptive CPE algorithms can also affect the performance of the dispersion compensation^15^,^16^,^31^. It has been indicated that laser phase noise can be converted into amplitude noise by chromatic dispersion^23^,^24^. Taking scenario (a) in Fig. 1 as an example, the phase noise from transmitter laser passes through both the transmission fibre and the EDC module, so the net experienced dispersion is close to zero. The phase noise from the LO laser only goes through the EDC module in the receiver, which is heavily dispersed in systems without any optical dispersion compensation. Thus, the LO phase noise will interplay with the EDC module to cause the effect of phase noise to amplitude noise conversion, and the induced EEPN will significantly affect the performance of long-haul high speed optical transmission systems^25^,^26^,^27^,^28^,^29^,^30^,^31^,^32^,^33^,^34^. Our previous work has also demonstrated that the EEPN arises from the non-zero net dispersion experienced by the laser phase noise, either from the transmitter laser or the LO laser^39^,^40^. Therefore, the EEPN originates from the interaction between the LO laser phase noise and the EDC (or the DBP) module in case (a), or from the interplay between the transmitter laser phase and the fibre dispersion in case (b). According to the different origins of EEPN, the effect can be categorized into equalization enhanced LO phase noise (EELOPN) for case (a) and equalization enhanced transmitter phase noise (EETxPN) for case (b).

The representations of the EELOPN (*E*_*EELOPN*_(*t*)) and the EETxPN (*E*_*EETxPN*_(*t*)) in the time-domain can be described by the following equations,


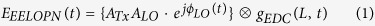



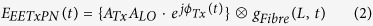


where *A*_*Tx*_ and *A*_*LO*_ are the amplitudes of the transmitter laser carrier and the LO laser optical wave respectively, *ϕ*_*Tx*_(*t*) and *ϕ*_*LO*_(*t*) are the phase fluctuation in the transmitter laser and the LO laser respectively, *g*_*EDC*_(*L*, *t*) is the time-domain transfer function of the electronic dispersion compensation filter, *g*_*Fibre*_(*L*, *t*) is the time-domain transfer function of the fibre, *L* is the fibre length, *t* represents the temporal variable, and ⊗ indicates the convolution operation.

Taking scenario (a) as an example, the transfer function *G*_*EDC*_(*L*, *ω*) of the electronic dispersion compensation filter in the frequency-domain, which is the Fourier transform of the time-domain transfer function *g*_*EDC*_(*L*, *t*), can be expressed as follows^9^,^10^,


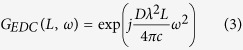


As shown Fig. 1(c), the phase of the EDC filter in Eq. (3) varies quadratically with frequency, which increases the gradient at a higher relative frequency (further from the central channel carrier frequency). For the same perturbation Δ*ω* in the frequency (e.g. the frequency shift due to the laser linewidth), the phase variation ΔΦ_2_ caused by the perturbation at a higher relative frequency is larger than the phase variation ΔΦ_1_ induced by the frequency perturbation at a lower relative frequency. Therefore, the interplay between the laser phase noise and the EDC module will be greater at a higher relative frequency, and will generate a more significant EEPN. Correspondingly, for the superchannel transmission systems, the outer sub-channels (e.g. channel 4 in Fig. 1(c)) will be impacted by more serious EEPN than the central sub-channel (e.g. channel 0 in Fig. 1(c)), when the dispersion compensation is applied over the entire superchannel simultaneously. It is clear then that, when only linear EDC is applied, it is beneficial to compensate only the intra-channel dispersion. However, it is a prerequisite of MC-DBP that the EDC is applied over all sub-channels simultaneously. The implications of this are investigated in more detail in the Results and Discussion sections.

## Results

### DP-64QAM superchannel transmission system

**Figure 1:**
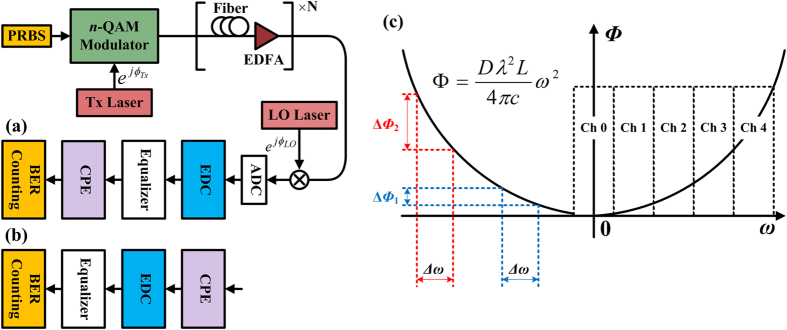
Schematic of EEPN in optical fibre communication system using electronic dispersion compensation (or digital back-propagation) and carrier phase estimation. (**a**) shows the DSP scenario for carrier phase estimation applied after CD compensation; (**b**) shows the DSP scenario for carrier phase estimation applied prior to CD compensation; (**c**) shows the phase variation due to frequency perturbation in dispersion compensation in EDC (or MC-DBP). (PRBS: pseudo random bit sequence, EDFA: erbium doped fibre amplifier, ADC: analog-to-digital convertor, BER: bit-error-rate, Ch: channel).

**Figure 2:**
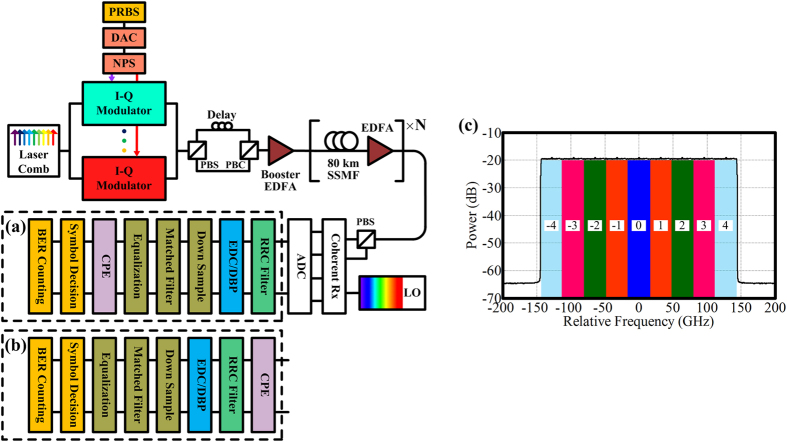
Schematic of 9-channel DP-64QAM superchannel transmission system using the EDC or the MC-DBP. (**a**) shows the DSP scenario for carrier phase estimation applied after EDC (or MC-DBP); (**b**) shows the DSP scenario for carrier phase estimation applied prior to EDC (or MC-DBP); (**c**) shows the simulated transmission spectrum and the schematic of back-propagated bandwidth in the 9-channel DP-64QAM superchannel transmission system, where the frequency 0 Hz refers to the superchannel central frequency (wavelength of 1550 nm). (PRBS: pseudo random bit sequence, PBS: polarization beam splitter, PBC: polarization beam combiner, ADC: analogue-to-digital convertor)

The setup of the 9-channel 32-Gbaud DP-64QAM superchannel transmission system is schematically illustrated in Fig. 2, and all numerical simulations were carried out using the split-step Fourier algorithm to solve the nonlinear Schrödinger equation with a digital resolution of 32 sample/symbol. In the transmitter, a 9-line 32-GHz spaced laser comb (centered at 1550 nm) is used as the phase-locked optical carrier for each sub-channel. Digital-to-analog convertor (DAC) with a resolution of 16-bit (to ensure no back-to-back implementation penalty) and root-raised-cosine (RRC) filter with a roll-off of 0.1% were used for the Nyquist pulse shaping (NPS). The transmitted symbol sequences were decorrelated with a delay of 256 symbols using a cyclical time shift to emulate the independent data transmission in each sub-channel and the sequences in each polarization were also decorrelated with a delay of half the sequence length. The standard single-mode fibre (SSMF) is simulated based on the split-step Fourier method with a step size of 0.1 km, and the detailed parameters are: span length of 80 km, attenuation coefficient of 0.2 dB/km, CD coefficient of 17 ps/nm/km, nonlinear coefficient of 1.2 /W/km. The dispersion slope and PMD effect were neglected. The noise figure of the erbium doped fibre amplifier (EDFA) was set to 4.5 dB. At the receiver, the received signal was mixed with a free-running LO laser and sampled at 32 samples/symbol, without any bandwidth limitation, which allows an ideal and synchronous detection of all the in-phase and quadrature signal components over the whole superchannel bandwidth. The DSP modules include a RRC filter for selecting the MC-DBP bandwidth, followed by MC-DBP (or linear EDC), down-sampling (to 2 samples/symbol), matched filter, multiple modulus algorithm equalization, ideal CPE, symbol de-mapping, and bit-error-rate (BER) measurement. The ideal CPE was realized by using the conjugate multiplication between the received signal and laser carrier phase, to isolate the influence of EEPN from the intrinsic laser phase noise. DSP details are described in Methods section. Corresponding to Fig. 1, the same two scenarios are considered in the simulations: (a) ideal CPE applied after the EDC/MC-DBP, (b) ideal CPE applied prior to the EDC/MC-DBP, as shown in Fig. 2(a,b), respectively. The spectrum of the 9-channel DP-64QAM coherent transmission system is shown in Fig. 2(c), where the number represents the sub-channel within the superchannel.

### Performance of MC-DBP in superchannel transmission

**Figure 3:**
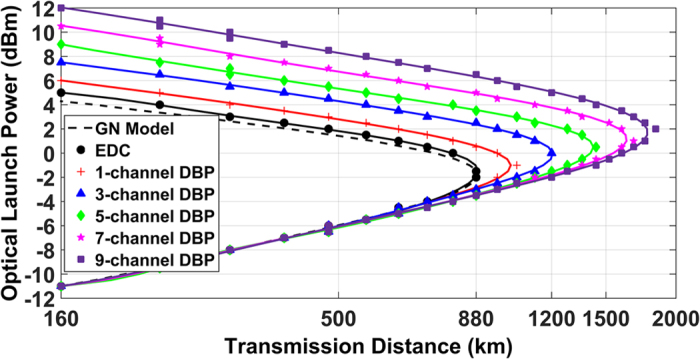
Maximum reach distance as a result of different optical launch powers at BER of 1.5 × 10^−2^ in the 9-channel DP-64QAM superchannel transmission system using the EDC and the MC-DBP. The markers are the simulation data, and the solid line is the 5^th^ order polynomial fit.

In all simulations, the MC-DBP algorithm was operated with the 800 steps per span and the nonlinear coefficient of 1.2 /W/km to ensure optimum operation of nonlinear compensation. Details of the optimization of MC-DBP are discussed in Methods section. The performance of the MC-DBP algorithm was investigated for the case in which the linewidths of both the transmitter and the LO lasers were set to 0 Hz to remove the influence from phase noise and EEPN. The achievable transmission distance for different launch powers for the central sub-channel (channel index of 0 in Fig. 2(c)) in the 9-channel DP-64QAM Nyquist transmission system is shown in Fig. 3, where the BER threshold was set to be 1.5 × 10^−2^ (Q^2^ factor of ~6.73 dB), corresponding to a 20% overhead hard-decision forward error correction (FEC) error-free BER threshold^41^. These results were obtained using either EDC or MC-DBP with different back-propagated bandwidths. It can be seen that when EDC only is applied, the maximum transmission distance is 880 km (11 SSMF spans) at the optimum launch power (−2 dBm). Single-channel DBP enhances the transmission distance by 18.2% (1040 km at launch power of −1 dBm), while the 9-channel (full-bandwidth) DBP results in 109% increase in the transmission distance (1840 km at launch power of 2 dBm). Effective improvements in the performance of the superchannel transmission system can be seen with each increment in the MC-DBP bandwidth. In addition, the analytical prediction of the transmission performance was also carried out using the perturbative Gaussian noise model in the frequency domain^42^,^43^, and good agreement can be seen between the analytical prediction (black dash) and the simulated reach curve with EDC only (black circles).

**Figure 4:**
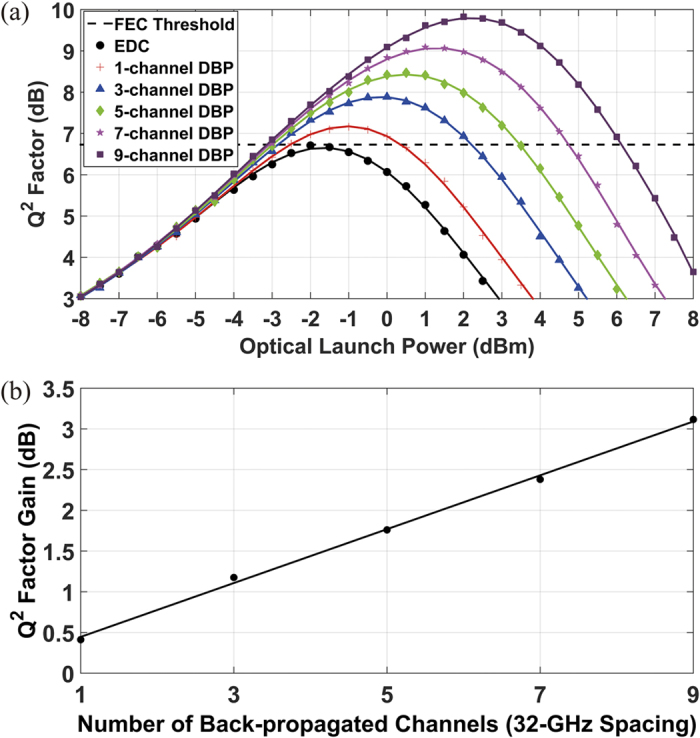
Performance of Q^2^ factor at 880 km (11 spans) SSMF in the 9-channel DP-64QAM transmission system using the MC-DBP over different back-propagated bandwidth. (**a**) shows Q^2^ factor versus different optical launch power for different number of back-propagated sub-channels; (**b**) shows Q^2^ factor gain at optimum launch power (compared to EDC-only case) for different numbers of back-propagated sub-channels. The markers are the simulation data, and the solid lines are the 5^th^ order polynomial fits in (**a**), and the linear fit in (**b**), respectively.

The performance of the MC-DBP in the Nyquist-spaced 9-channel DP-64QAM superchannel transmission system has also been investigated in terms of Q^2^ factor for different optical launch powers, as shown in Fig. 4. The transmission distance is the maximum achievable distance −880 km (11 SSMF spans) - for the system using EDC only. All the Q^2^ factors are converted directly from the measured BER values. It can be seen from Fig. 4(a) that the performance of the MC-DBP in the superchannel transmission system improves with the increment of the back-propagated bandwidth, and the improvement will not be saturated in the ideal operation of the MC-DBP. The best achievable Q^2^ factor with EDC only is ~6.73 dB at an optimum launch power of −2 dBm. When the single-channel DBP is employed for SPM compensation, the best achievable Q^2^ factor was improved to ~7.2 dB at an optimum launch power of −1 dBm. When the 9-channel (full-bandwidth) DBP is applied over the entire superchannel for compensating the SPM, the XPM and the FWM simultaneously, the best achievable Q^2^ factor is improved up to ~9.8 dB at the optimum launch power of 2 dBm. Compared to the optimum Q^2^ factor in the EDC-only case, the improvement in the best achieved Q^2^ factor (at each optimum launch power) for different number of back-propagated sub-channels, which can be defined as the Q^2^ factor gain (in dB), is illustrated in Fig. 4(b), in which a linear fit has been used to show the trend. The Q^2^ factor gain (in dB) increases linearly with the increment of the number of back-propagated sub-channels, and the Q^2^ factor gain of the 9-channel (full-bandwidth) DBP is ~3.1 dB compared to the EDC-only case, and is ~2.6 dB compared to conventional single-channel DBP case. This investigation on the operation and the optimization of the MC-DBP gives a basis for the optimal operation of the full-bandwidth DBP and a benchmark for the evaluation of the 9-channel DP-64QAM Nyquist superchannel transmission system for the ideal case of Tx and LO lasers with zero linewidth.

### Influence of EEPN in superchannel transmission using EDC and MC-DBP

The performance of all the sub-channels in the 9-channel DP-64QAM superchannel transmission system was investigated in terms of Q^2^ factors (converted directly from BERs), evaluated both using the EDC and the 9-channel (full-bandwidth) DBP. Corresponding to previous descriptions, two DSP scenarios were considered to assess the origin and the impact of EEPN: (a) CPE implemented after the DBP/EDC, (b) CPE implemented prior to the DBP/EDC.

**Figure 5:**
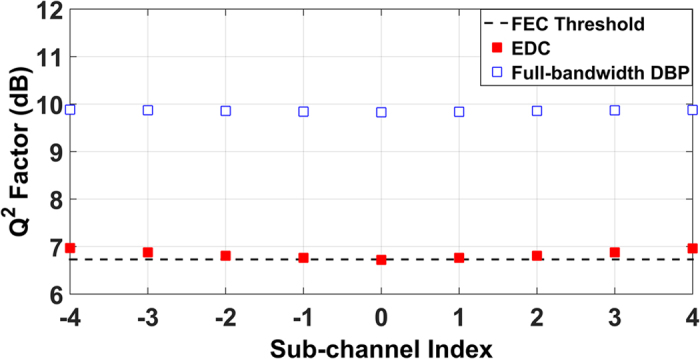
Performance of each sub-channel in the 9-channel DP-64QAM transmission system without any EEPN, both the Tx laser and the LO laser linewidths are 0 Hz. The FEC threshold is again the Q^2^ factor of ~6.73 dB (BER of 1.5 × 10^−2^), corresponding to the 20% overhead hard-decision FEC error-free threshold.

The Q^2^ factor of each sub-channel in the Nyquist spaced 9-channel DP-64QAM optical transmission system without any influence of EEPN (zero linewidth for both the Tx and the LO lasers) is illustrated in Fig. 5, in which the results were obtained both using the EDC and the full-bandwidth DBP. The optical launch power in all the sub-channels is −2 dBm for the EDC case, and the optical launch power in all the sub-channels is 2 dBm for the full-bandwidth DBP case, which corresponds to the optimum launch powers for the central sub-channel using EDC and full-bandwidth DBP, respectively. It was found in Fig. 5 that the central sub-channel gives a slightly worse performance than the outer sub-channels in the EDC-only case, since the central sub-channel experiences more significant nonlinear interference than the outer sub-channels due to the inter-channel nonlinearities (XPM and FWM). For the case of full-bandwidth DBP, all sub-channels exhibit almost the same Q^2^ factor behavior, since the dominant nonlinearities (signal-signal interactions within the bandwidth of nonlinear compensation) have been removed.

**Figure 6:**
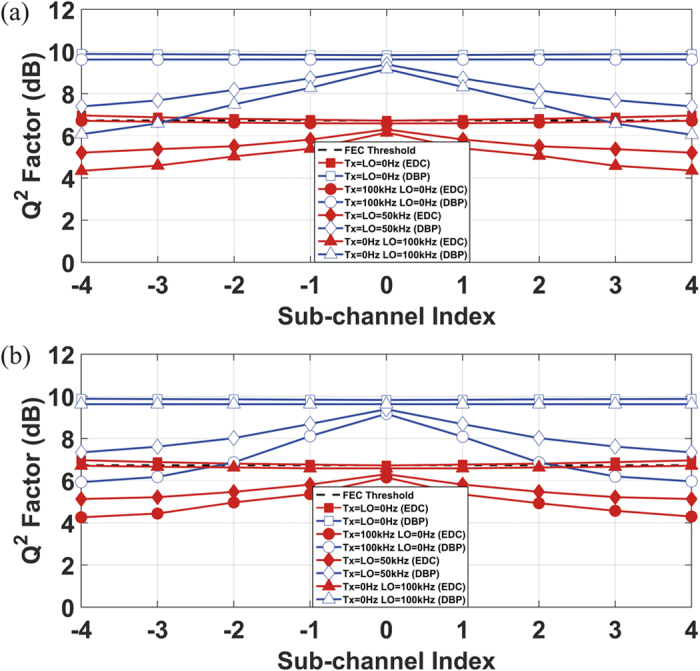
Performance of each sub-channel in Nyquist-spaced 9-channel DP-64QAM transmission system influenced by EEPN. The simulation is carried out under different distributions of the Tx and the LO laser linewidths, and both the EDC and the DBP have been applied over the whole superchannel. (**a**) shows the scenario for ideal CPE implemented after EDC/DBP; (**b**) shows the scenario for ideal CPE implemented before EDC/DBP.

However, in the practical long-haul superchannel transmission system where a non-zero linewidth of the Tx and the LO lasers exists, the impact of EEPN should be considered. As described above, due to the larger phase slope for the outer sub-channels in the dispersion compensation filter (or the fibre dispersion) transfer function, the outer sub-channels will have a more significant EEPN than the central sub-channel, which will generate a more serious distortion. Therefore, the side sub-channels will exhibit a worse performance than the central sub-channel due to the more significant EEPN. The performance of all the sub-channels in the Nyquist-spaced 9-channel DP-64QAM superchannel system with a transmission distance of 880 km (11 fibre spans) is illustrated in Fig. 6, in which a significant EEPN has been applied. Again, two DSP scenarios are considered in the numerical simulations: Fig. 6(a) shows results for the ideal CPE realized after the DBP/EDC, and Fig. 6(b) shows the results for the ideal CPE implemented prior to the DBP/EDC. To investigate the origin and the impact of EEPN, different distributions of the linewidths from the Tx laser and the LO laser have been applied: (1) the Tx laser linewidth is 100 kHz, and the LO laser linewidth is 0 Hz, (2) both the Tx laser and the LO laser linewidths are 50 kHz, (3) the Tx laser linewdith is 0 Hz, and the LO linewidth is 100 kHz. It can be found in Fig. 6(a) that the EEPN originates from the interaction between the LO laser phase noise and the EDC/DBP module, corresponding to the case of equalization enhanced LO phase noise. With the increment of the LO laser linewidth (from 0 Hz to 100 kHz) in Fig. 6(a), the outer sub-channels exhibit a significantly worse performance than the central sub-channel due to the EEPN induced additional noise, in both cases of the EDC and the full-bandwidth DBP. When the Tx laser linewidth is 100 kHz and the LO laser linewidth is 0 Hz, the performance of all the sub-channels shows a similar behavior, and is almost the same as for the ideal case (both Tx and LO laser linewidths are 0 Hz). This means that there is no EEPN influence in such scenario, since the interplay between the Tx laser phase noise and fibre dispersion can be fully compensated either by EDC or DBP. The performance of all the sub-channels in the superchannel transmission system in Fig. 6(b) is exactly reversed compared to Fig. 6(a), where the EEPN arises from the interaction between the Tx laser phase noise and fibre dispersion, corresponding to the case of equalization enhanced Tx phase noise.

**Figure 7:**
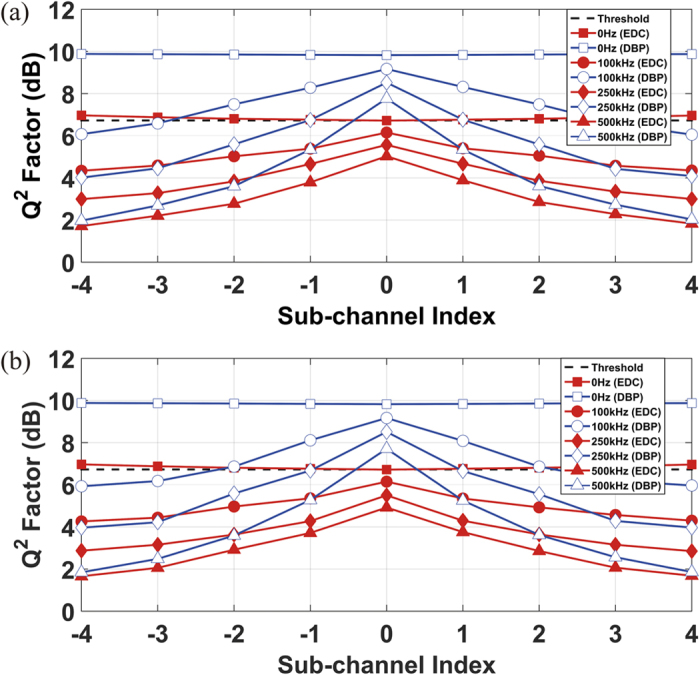
Performance of each sub-channel in Nyquist-spaced 9-channel DP-64QAM transmission system influenced by EEPN, where both the EDC and the DBP have been applied over the whole superchannel. (**a**) shows the CPE implemented after the EDC/DBP, the Tx laser linewidth is 0 Hz and the indicated linewidth is for the LO laser, (**b**) shows the CPE implemented before the EDC/DBP, the LO laser linewidth is 0 Hz and the indicated linewidth is for the Tx laser.

Figure 7 shows the degradation in the performance of all the sub-channels in the 9-channel DP-64QAM transmission system with the increment of the EEPN, where an increase in the Tx or the LO laser linewidths is applied. The transmission distance is again 880 km (11 fibre spans). In Fig. 7(a), the Tx laser linewidth is kept at 0 Hz and the LO laser linewidth is varied from 0 to 500 kHz. In Fig. 7(b), the LO laser linewidth is kept at 0 Hz and the Tx laser linewidth is varied over the range from 0 to 500 kHz. It can be found that, in both cases using the EDC and the full-bandwidth DBP, the performance of the transmission system is significantly degraded by EEPN with the increment of the laser linewidth, and the performance of the outer sub-channels behaves worse than the central sub-channel with the increment of the laser linewidth, due to the more severe EEPN induced from the quadratic phase distribution of the EDC (or the fibre dispersion) transfer function. It is also found that the EEPN rises with the increment of the LO laser linewidth in Fig. 7(a) since the EEPN originates from LO laser phase noise for the case of CPE implemented after the EDC/DBP, and the EEPN rises with the increment of the Tx laser linewidth in Fig. 7(b) since the EEPN originates from Tx laser phase noise for the case of CPE implemented prior to the EDC/DBP. In addition, it has also been verified in our simulation that, to ensure that the Q^2^ factor in all the sub-channels is above the 20% overhead hard-decision FEC error-free BER threshold (1.5 × 10^−2^) in the full-bandwidth digital back-propagation scheme, the EEPN puts a limitation on the maximum tolerable linewidth (~60 kHz for our system) on the LO laser in the case of CPE implemented after DBP, and the Tx laser in the case of CPE implemented before DBP.

## Discussion

It is worth noting that the dispersion compensation in both the EDC and the full-bandwidth DBP were applied over the entire superchannel bandwidth in all the above analysis. In the forward propagation along the fibre, the phase delay in each sub-channel is different, since the fibre dispersion profile (quadratic shape) is different in different frequency bands. Thus, the carrier phase will have different delays in the fibre for different sub-channels. In the receiver, if the dispersion is compensated over the entire superchannel simultaneously, the EDC filter will have the same dispersion profile as the fibre over the superchannel bandwidth, and thus the carrier phase in all sub-channels will be synchronized, and the phase noise can be compensated perfectly by applying the conjugate multiplication with the original carrier phase. According to the above investigations, the outer sub-channels will suffer more pronounced EEPN than the central sub-channel in such case. On the other hand, if the dispersion is compensated on an individual sub-channel basis (using a frequency shift and a matched filter applied before EDC), the EDC filter will be designed separately by only considering the frequency range within each sub-channel. Thus the overall phase response will be different from the fibre response over the whole superchannel bandwidth. The carrier phase after the EDC will have different delays in different sub-channels, and the phase noise in the outer sub-channels cannot be perfectly removed by applying the conjugate multiplication with the original carrier phase, which is only synchronized with the central sub-channel, and additional phase delays need to be considered in the ideal CPE. In this case, the impact of EEPN on the specific outer sub-channel can be reduced to some extent, and will be the same as that in the central sub-channel. Note that practical CPE algorithms work in both cases for compensating the intrinsic carrier phase noise, since the CPE algorithms only consider the current carrier phase noise in the measured sub-channel. However, as previously noted, the dispersion compensation in the MC-DBP (or full-bandwidth DBP) has to be applied simultaneously over the whole back-propagated sub-channels, since all the information of the nonlinear interference in the involved sub-channels is required for the MC-DBP to cancel both the intra-channel and the inter-channel nonlinearities. In this case, the EEPN in the outer sub-channels in Nyquist superchannel transmission systems cannot be reduced.

All the above numerical simulations have been implemented based on the 9-channel 32-Gbaud Nyquist spaced DP-64QAM optical transmission system. The findings can also be qualitatively applied to transmission systems using different modulation formats and different numbers of WDM sub-channels. The influence of the EEPN in superchannel transmission systems will be more severe for higher-order modulation formats and larger superchannel bandwidths. For systems employing a lower-order modulation format, such as QPSK and 16-QAM, the influence of the EEPN could be less significant. However, to achieve the same throughput, a higher symbol rate (transmission bandwidth) has to be used, for which the impact of EEPN will be stronger. Thus the impact of EEPN would still be significant for QPSK and 16-QAM superchannel systems with a high data rate. Meanwhile, the maximum reach can be improved considerably by using the full-bandwidth DBP over the whole superchannel system, in that case a more serious impact of EEPN should be considered due to a larger accumulated fibre dispersion.

Regarding the carrier phase estimation, an ideal CPE has been employed for the carrier recovery using the conjugate multiplication between the signal and the extracted intrinsic laser phase noise. This is to isolate the influence from EEPN, where no amplitude noise mitigation effect is employed in the CPE module. However, in practical DSP operation for the coherent detection, where some CPE approaches such as the block-wise average and the Viterbi-Viterbi algorithms are applied for compensating the laser phase noise^12^,^17^, the amplitude noise can also be mitigated to some extent by using the block-wise average and the Viterbi-Viterbi CPE algorithm with a large block size. The selection of the block size involves making a compromise between the additive amplitude noise and the phase noise^44^,^45^. The effect of EEPN in such practical systems could be less obvious than the transmission system using the ideal CPE discussed in this paper. However, the impact of EEPN is still considerable in the long-haul optical fibre transmission systems with high order modulation formats and large transmission bandwidths, when some practical CPE algorithms are applied.

EEPN originates from the interplay between the laser phase noise and the dispersion in the long-haul optical communication system using the EDC or the DBP, and significantly impacts the performance of the transmission system. The digital coherence enhancement based approaches can achieve an effective mitigation for the EEPN, while an independent measurement (or a complicated phase de-correlation) to estimate the LO laser phase fluctuation is necessary^34^,^46^,^47^. The EEPN can also be mitigated efficiently by applying a certain cut-off frequency to the LO laser, whereas a high-pass filtering based on electrical feedback or digital coherence enhancement is required to suppress the frequency noise^48^. On the contrary, there is no EEPN in the transmission systems employing optical dispersion compensation, e.g. using dispersion compensating fibres and chirped fibre Bragg gratings, while the suppression of fibre nonlinear effects in such systems becomes more critical. One applicable method can be the optical back-propagation approach, which optically compensates the chromatic dispersion and the nonlinearities simultaneously^49^,^50^. Thus both EEPN and fibre nonlinearities can be mitigated with a low complexity.

In addition to MC-DBP, the optical phase conjugation (OPC) is also a promising approach to compensate the fibre nonlinearities over multi-channel WDM transmission systems, where the phase conjugation of the transmitted signal is employed to generate an opposite nonlinear phase shift for compensating the nonlinear effects^51^,^52^. Since the optical dispersion compensation is always involved, there will be no EEPN from the transmitter and the LO lasers phase noise in the OPC based transmission systems. However, the phase noise of the high-power pump laser, which is used for implementing the FWM based phase conjugation, can be transferred into the phase of the output signal after the phase conjugation^53^. Thus the EEPN in the OPC based systems arises from the phase noise of the high-power pump laser, and the EEPN impact will depend on the linewidth of the pump laser and the transmission distance of the phase-conjugated signal.

## Methods

### Evaluation of EEPN induced noise

In both cases of the EELOPN and the EETxPN, the EEPN induced noise scales linearly with the accumulated CD, the linewidth of laser (either the transmitter or the LO), and the transmission bandwidth. For single-channel systems, the variance of the additional noise due to the EEPN can be expressed as^25^,^31^,


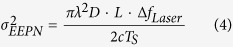


where λ is the central wavelength of the optical carrier, *c* is the light speed in vacuum, *D* is the CD coefficient of fibre, *L* is the fibre length, Δ*f*_*Laser*_ is the 3-dB linewidth (assuming a Lorentzian distribution) of the contributive laser (Δ*f*_*Tx*_ for the Tx laser or Δ*f*_*LO*_ for the LO laser), and *T*_*S*_ is the transmitted symbol period. Note that the analytical derivation of EEPN in Eq. (4) is also applicable for transmission systems employing electronic dispersion pre-compensation^40^, while it is not appropriate for any adaptive electronic dispersion compensation, since the adaptive dispersion compensation filter will interact with both the Tx laser and the LO laser phase noise simultaneously^54^.

### Digital signal processing in numerical simulation

In the DSP based receiver, the EDC was realized using a frequency domain equalizer^10^, and the MC-DBP was implemented using the reverse split-step Fourier solution of the signal nonlinear propagation based on the Manakov equation with a 0.5 split ratio of CD compensation^14^,^19^,^22^. The ideal RRC filter was applied to select the desired back-propagated bandwidth for the MC-DBP, and also to reject the unwanted out-of-band ASE noise. The back-propagated bandwidth was 32-GHz for the single-channel DBP, increasing to 288-GHz for 9-channel (full-bandwidth) DBP. The sampling rate was always kept to 32 samples/symbol in the EDC and the MC-DBP operations. The matched filter was used to select the sub-channel of interest and to optimize the signal-to-noise ratio of the processed signal. The multiple modulus algorithm with 21 taps was used for the polarization equalization and the residual impairments equalization. The CPE was realized using ideal phase estimation, which assumes that the exact carrier phase is known in the simulations. The ideal CPE was implemented by using the conjugate multiplication between the received signal and the intrinsic laser carrier phase. Finally, symbol de-mapping and bit-error-rate counting were carried out to assess the transmission performance of the measured sub-channel based on 2^18^ bits, with a pseudo random bit sequence (PRBS) pattern length of 2^15^−1.

### Optimization of MC-DBP algorithm

**Figure 8:**
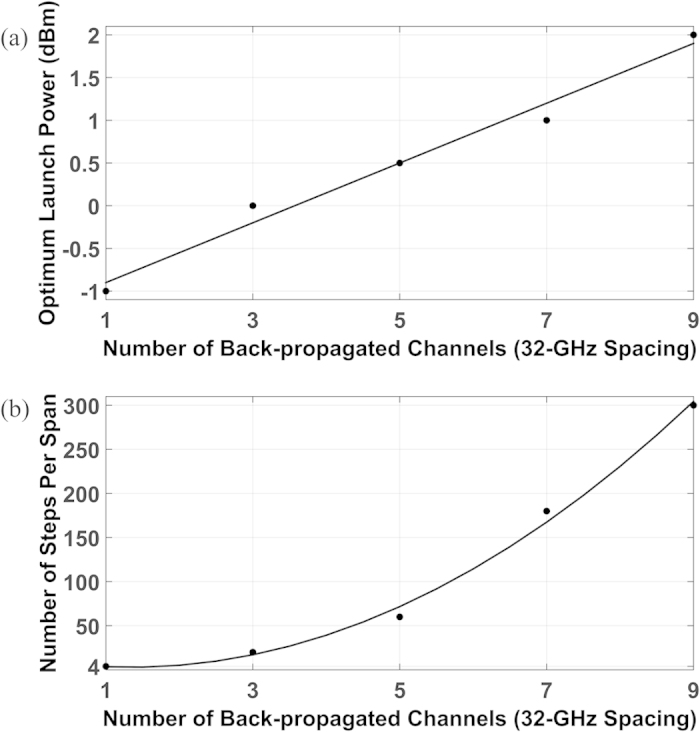
Optimization of MC-DBP for different numbers of back-propagated sub-channels. (**a**) shows the optimum optical launch power per sub-channel versus the number of back-propagated sub-channels in MC-DBP; (**b**) shows the minimum required number of steps per fibre span in MC-DBP versus the number of back-propagated sub-channels. The markers are the simulation data, and the solid lines are the linear fit in (**a**), and the second-order polynomial fit in (**b**), respectively.

The optimization of the MC-DBP algorithm was investigated for the case in which the linewidths of both the transmitter and the LO lasers were set to 0 Hz. To achieve the best performance, the operation of the MC-DBP over different back-propagated bandwidths was investigated in terms of the number of steps per fibre span *N*_*SPS*_, with a nonlinear coefficient parameter in the MC-DBP *γ*_*DBP *_= 1.2 /W/km (the same as the fibre nonlinear coefficient in the forward propagation). The transmission distance was again 880 km (11 SSMF spans). The optimum launch power was always selected to achieve the lowest BER in the central sub-channel for the particular MC-DBP bandwidth used, as shown in Fig. 8(a). It can be found that the optimum launch power per sub-channel varies from −1 dBm for single-channel DBP, up to 2 dBm for 9-channel (full-bandwidth) DBP. The minimum required number of steps per fibre span for different numbers of back-propagated channels, which is defined as the minimum number of steps to achieve the best Q^2^ factor in the central sub-channel for the MC-DBP, is illustrated in Fig. 8(b), where it can be seen that, for single-channel DBP (32-GHz), the minimum number of steps per span is about 4, increasing to 300 for 9-channel (full-bandwidth) DBP. The good agreement between the minimum number of steps per span and the polynomial fit shows that the required number of steps per span increases quadratically with the number of back-propagated sub-channels.

The optimization and the operation of MC-DBP for compensating the fiber nonlinearities have also been investigated in our previous work^55^, where the performance of MC-DBP has been assessed experimentally based on a 7-channel 10-Gbaud DP-16QAM superchannel transmission system. In that work, the required number of steps per span in the MC-DBP algorithm was investigated and a similar trend of an increase with the increment of back-propagated bandwidth was observed. However, the details, such as the required number of steps per span, the Q^2^ factor gain and maximum reach gain (compared to the EDC-only case), are different in the systems using different modulation formats, different numbers of channels, and different symbol rates^22^,^55^,^56^. Therefore, the optimization and the investigation of the MC-DBP algorithm herein provide a specific benchmark for the operation and the performance of MC-DBP in a Nyquist-spaced 9-channel 32-Gbaud DP-64QAM superchannel transmission system.

### Perturbative Gaussian noise model

In the long-haul dispersion-uncompensated transmission link, the performance of the system is limited by the ASE noise from EDFA and the nonlinear distortions from the Kerr effect in the fibre. It has been demonstrated that the nonlinear interference in the dispersion-unmanaged WDM transmission system can be approximately regarded as an additive noise with a zero-mean Gaussian distribution, statistically independent from ASE noise^42^,^43^. Based on the Gaussian noise model, the optical signal-to-noise ratio (OSNR) to determine the bit-error-rate in the transmission system can be expressed as.


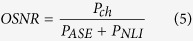


where *P*_*ch*_ is the optical launch power per channel, *P*_*ASE*_ is the linear ASE noise of the EDFA, and *P*_*NLI*_ is the nonlinear distortions from the fibre Kerr effects. The ASE noise in the EDFA can be described by the following equation^42^,^43^,


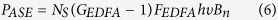


where *N*_*S*_ is the number of fibre spans, *G*_*EDFA*_ is the gain of EDFA, *F*_*EDFA*_ is the noise figure of EDFA, *h* is the Planck constant, *ν* is the central frequency of the optical wave, and *B*_*n*_ is the noise bandwidth. For the 0.1 nm OSNR, *B*_*n *_≈ 12.5 GHz is the reference noise bandwidth.

For the Nyquist-spaced WDM superchannel system, the nonlinear interference among the transmitted signals can be expressed in a closed-form formula^42^,


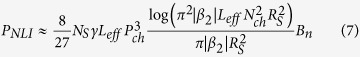


where *N*_*S*_ is the number of fibre spans, *γ* is the nonlinear coefficient of fibre, *β*_*2*_ is the fibre dispersion parameter, *L*_*eff*_ is the effective length of the fibre, *N*_*ch*_ is the number of WDM sub-channels, *R*_*S*_ is the symbol rate of transmitted signal, and *B*_*n*_ is the noise bandwidth. By using Eq. (6) and Eq. (7), Eq. (5) can give an evaluation of the OSNR in the Nyquist-spaced superchannel system without any nonlinear compensation.

## Additional Information

**How to cite this article**: Xu, T. *et al.* Equalization enhanced phase noise in Nyquist-spaced superchannel transmission systems using multi-channel digital back-propagation. *Sci. Rep.* **5**, 13990; doi: 10.1038/srep13990 (2015).
